# Rapamycin Preserves Neural Tissue, Promotes Schwann Cell Myelination and Reduces Glial Scar Formation After Hemi-Contusion Spinal Cord Injury in Mice

**DOI:** 10.3389/fnmol.2020.574041

**Published:** 2021-01-22

**Authors:** Junhao Liu, Ruoyao Li, Zucheng Huang, Junyu Lin, Wei Ji, Zhiping Huang, Qi Liu, Xiaoliang Wu, Xiuhua Wu, Hui Jiang, Yongnong Ye, Qingan Zhu

**Affiliations:** ^1^Division of Spine Surgery, Department of Orthopaedics, Nanfang Hospital, Southern Medical University, Guangzhou, China; ^2^Division of Spine Surgery, Department of Orthopaedics, Guangzhou First People’s Hospital, School of Medicine, South China University of Technology, Guangzhou, China; ^3^Pharmaceutical Department, Guangdong Provincial Hospital of Traditional Chinese Medicine, Guangzhou, China

**Keywords:** autophagy, spinal cord injury, rapamycin, Schwann cells, neuroprotection

## Abstract

Protecting white matter is one of the key treatment strategies for spinal cord injury (SCI), including alleviation of myelin loss and promotion of remyelination. Rapamycin has been shown neuroprotective effects against SCI and cardiotoxic effects while enhancing autophagy. However, specific neuroprotection of rapamycin for the white matter after cervical SCI has not been reported. Therefore, we aim to evaluate the role of rapamycin in neuroprotection after hemi-contusion SCI in mice. Forty-six 8-week-old mice were randomly assigned into the rapamycin group (*n* = 16), vehicle group (*n* = 16), and sham group (*n* = 10). All mice of the rapamycin and vehicle groups received a unilateral contusion with 1.2-mm displacement at C5 followed by daily intraperitoneal injection of rapamycin or dimethyl sulfoxide solution (1.5 mg⋅kg^–1^⋅day^–1^). The behavioral assessment was conducted before the injury, 3 days and every 2 weeks post-injury (WPI). The autophagy-related proteins, the area of spared white matter, the number of oligodendrocytes (OLs) and axons were evaluated at 12 WPI, as well as the glial scar and the myelin sheaths formed by Schwann cells at the epicenter. The 1.2 mm contusion led to a consistent moderate–severe SCI in terms of motor function and tissue damage. Rapamycin administration promoted autophagy in spinal cord tissue after injury and reduced the glial scar at the epicenter. Additionally, rapamycin increased the number of OLs and improved motor function significantly than in the vehicle group. Furthermore, the rapamycin injection resulted in an increase of Schwann cell-mediated remyelination and weight loss. Our results suggest that rapamycin can enhance autophagy, promote Schwann cell myelination and motor function recovery by preserved neural tissue, and reduce glial scar after hemi-contusive cervical SCI, indicating a potential strategy for SCI treatment.

## Introduction

Spinal cord injury (SCI) is a devastating disease that can lead to permanent motor and sensory dysfunction. Except for protecting the remaining neurons in the spinal cord, more and more attention has been paid to the treatment strategies for protecting white matter. The protection of white matter is mainly to reduce myelin loss and promote remyelination in the acute and chronic SCI phases, respectively. Normally, the myelin sheaths in the spinal cord are mainly formed and maintained by oligodendrocytes (OLs) in the central nervous system (CNS). Inflammation and peroxidation in the secondary pathology following SCI often contributed to the death of OLs (Connor and Menzies, 1996; Thorburne and Juurlink, 1996; Almad et al., 2011) and then caused the loss of myelin sheath. The myelin loss can cause abnormal transmission of electrical signals and degeneration of the axons, which will further aggravate the motor and sensory dysfunction. Therefore, alleviating myelin loss and promoting endogenous remyelination are an important strategy for the treatment of SCI.

Rapamycin was commonly used to induce autophagy by inhibiting the mTOR signaling pathway (Mizushima et al., 2008). The administration of rapamycin had multiple effects for promoting functional recovery after SCI, including anti-inflammation (Chen et al., 2013; Tateda et al., 2017), reduction of glial scar formation and neural tissue damage (Sekiguchi et al., 2012; Chen et al., 2016), and neuroprotection (Wang et al., 2014). Saraswat Ohri et al. (2018) suppressed autophagy by knocking out the ATG5 gene in OLs, further demonstrating that the functional recovery can be limited by blocking autophagy in OLs after SCI. In addition, LC3 and Beclin-1, the characteristic markers of autophagy, were positive around the terminal deoxynucleotidyl transferase dUTP nick end labeling (TUNEL)-positive cells and contributed to the neural tissue damage after SCI (Kanno et al., 2009, 2011). Furthermore, Norrmén et al. (2018) reported that the mTORC1 played an important role in clearing myelin properly and dedifferentiating Schwann cells (SCs) after nerve injury. Despite that mTOR has gained interest as a mediator of plasticity, regeneration, and nociceptor hypersensitivity in the injured spinal cord, caution against the use of rapamycin as a therapeutic intervention for SCI has been reported recently because it evokes toxic weight loss and exacerbates cardiovascular dysfunction (Eldahan et al., 2018). However, the specific neuroprotective effect of rapamycin in the white matter after cervical SCI has not been reported.

Therefore, we aim to evaluate the role of rapamycin in neuroprotection after SCI. In the present study, the neuroprotection of rapamycin was observed in an innovative hemi-contusion SCI model. Behavioral and histological analyses were used to assess the therapeutic effect after injury. The area of spared white matter and glial scar were assessed at 12 weeks post-injury (WPI), as well as OLs and SCs of the spinal cord tissue at the epicenter.

## Materials and Methods

### Experimental Design

A total of *n* = 42 female C57 mice (8 weeks old) purchased from the Laboratory Animal Center at Southern Medical University were used in the present study. The animal study was reviewed and approved by the Experimental Animal Welfare and Ethics Committee of Southern Medical University, and all animal procedures are implemented following the Public Health Service Policy on Humane Care and Use of Laboratory Animals, Guide for the care and use of laboratory animals (Institute of Laboratory Animal Resources, National Research Council, 1996). Animals were randomly assigned to the rapamycin-treated group (*n* = 16), vehicle-treated group (*n* = 16), and sham group (*n* = 10). All animals were sacrificed at 12 weeks after SCI. During this time, behavioral experiments were performed to evaluate the degree of motor function recovery in the SCI animals.

### Hemi-Contusion Spinal Cord Injury

Mice were first anesthetized with 3% isoflurane (R510–22, RWD Biomed Inc., Shenzhen, China), and then anesthesia was maintained with 1.5–2% isoflurane during surgery. The mice were placed in a prone position, and the neck and back were shaved carefully and then disinfected with iodine and alcohol. A dorsal midline incision was performed on the neck, and the skin and muscles were separated to expose the C4–C6 lamina. A partial laminectomy was performed at C5 to expose the left side of the spinal cord, and then a homemade spinal clamp was used to stabilize the vertebral column of C4–C6. The impactor head (*ϕ* = 1 mm) was lowered slowly until it came into contact with the dura, and the contusion displacement and speed were set at 1.2 mm and 300 mm/s, respectively. After contusion, the incision was sutured layer by layer and then disinfected by iodine. The mice were transferred to a thermostat at a constant temperature of 32°C until they awoke.

### Rapamycin Administration

Rapamycin (R8140, Solarbio, Peking, China) was dissolved in dimethyl sulfoxide (DMSO; ST038, Beyotime, Shanghai, China) at 25 mg/ml before administration. In the rapamycin-treated group, animals have received rapamycin at a dose of 1.5 mg/kg per day after injury through intraperitoneal injection, with control animals received DMSO only. All the injured mice were administrated by rapamycin or DMSO until 12 WPI.

### Behavioral Assessment

All animals were included in the behavioral assessment, and the assessments were evaluated by two independent observers. To increase the activity of the mice, the behavioral assessment was performed during the dark cycle. Behavioral assessment including rearing and grooming, which had been reported previously, was conducted (Bertelli and Mira, 1993; Liu et al., 1999; Schallert et al., 2000; Gensel et al., 2006). The baseline values were established before surgery, and the behavioral assessment was conducted before injection at each time, at 3 days post-injury (DPI), and then every 2 weeks after surgery until the termination of the experiment.

#### Rearing Test

The mice were placed in a transparent cylinder with a diameter of 20 cm and videotaped for 15 min before stopped. The first 20 climbing moves (left forelimb touch, right forelimb touch, and both forelimbs touch) or all the climbing moves within 15 min were analyzed for each animal. Then the usage rate of the ipsilateral forelimb during this process was calculated. The test was performed on day 3 and every 2 weeks post-surgery.

#### Grooming Test

This test was mainly used to evaluate the motor function of the elbow and shoulder. The scoring system, which came from Bertelli’s study, was as follows: 1 point, animals’ foreclaws can touch the area under their mouth; 2 points, their foreclaws can touch the area between their mouth and eyes; 3 points, the claws can reach the eyes; 4 points, the claws can reach the front of the ear, but not the back; and 5 points, the claws can go behind the ears (Bertelli and Mira, 1993). This assessment was based on a 10 min video recording for each animal. Baseline scores were established preoperatively, and then the animals were tested on day 3 and every 2 weeks post-surgery.

### Perfusion and Tissue Processing

For histological analysis, animals were subjected to deep anesthesia with sodium pentobarbital and then perfused transcardially with 20 ml of phosphate-buffered saline (PBS) and followed by 40 ml of 4% paraformaldehyde (PFA; P6148, Biotopped Life sciences, Beijing, China) at 12 WPI. After identification of the location of the lesion site, the cervical spinal cord from C1 to C8 was intercepted. The spinal cords were post-fixed with PFA overnight and then cryoprotected at 4°C in ascending sucrose solution (12, 18, and 24%, in PBS solution). Samples were embedded in optimal cutting temperature (OCT) compound (Tissue-Tek, 4583, SAKURA) and stored at −80°C until use. Five to six samples in the injured groups and four samples in the sham group were sliced transversely into 20 μm using a cryostat (Cryotome^TM^ FSE, Thermo Fisher Scientific, United States), and three samples in the injured group were sliced longitudinally into 20 μm. For the cross sections, 10 slices were stored at −80°C, and the intervals of each section were 200 μm.

For Western blot (WB), mice (four in each group) were transcardially perfused with 20 ml of iced PBS only. The spinal cord tissue (5 mm) centered on the lesion site was removed on the ice. The samples were labeled and transferred quickly to be stored at −80°C for processing.

### Eriochrome Cyanine Staining

The slices were allowed to air dry at room temperature (RT) and then treated with xylene to wash out the OCT compound and a graded ethanol series (100, 90, 70, and 50%) for rehydration. Next, slices were washed with clean water to remove the ethanol. Slices were placed in eriochrome cyanine (EC) solution for 10 min followed by two rinses with clean water. After washing, the slices were subjected to differentiation with NH_4_OH:H_2_O (1:6) until there was a distinct demarcation between white matter and gray matter. Finally, after dehydration with gradient ethanol and permeabilization with xylene, slices were cover-slipped with neutral gum. Optical microscopic examination was performed when the slices were completely dry.

### Immunohistochemistry

The slices were left to dry at RT for 1 h and washed once with 0.01 M of PBS. In order to stain myelin effectively, slices were placed in ascending (50, 70, 90, 95, and 100%) and then descending ethanol dilutions (100, 95, 90, 70, and 50%). Again, the slices were washed with 0.01 M of PBS and subsequently blocked in 10% normal donkey serum (NDS) dissolved in 0.01 M of PBS with 0.1% Triton X-100 for 30 min. Primary antibodies diluted by 0.01 M of PBS with 0.1% Triton X-100 were applied to the sections overnight. The next morning, slices were washed with PBS and then incubated by secondary antibodies (Alexa Fluorescence 488, 594, and 647, Abcam, United Kingdom) diluted by 0.01 M of PBS with 0.1% Triton X-100 for 2 h. The slices were washed again and cover-slipped using Fluoroshield (ab104135, Abcam, United Kingdom). The antibodies used were directed against the following antigens: glial fibrillary acidic protein (GFAP) (1:1,000, Cell Signaling Technology, 3,670), myelin basic protein (MBP) (1:500, Abcam, ab4039), SMI312 (1:1,000, Covance, SMI-312R-100), NeuN (1:800, Abcam, ab177487), P0 (1:200, Abcam, ab31851), CC1 (1:300, Abcam, ab16794), and Olig2 (1:500, Millipore, AB9610).

### Histological Analysis

A Leica microscope (DM4000; Leica), a Zeiss confocal microscope (LM880, Zeiss, Germany), and Zen 2 software (Version 3.0, Zeiss, Germany) were used for taking images and analysis. The analysis was performed by Zucheng Huang and Ruoyao Li separately in a blinded manner. For analysis of the area of spared white matter, the sections stained with EC were taken at 100× magnification, including the epicenter and 0.2 and 0.4 mm rostral and caudal. Additionally, for analysis of the area of spared white matter and spared tissue, cross sections of the whole spinal cord stained with MBP/GFAP were taken at 100 × magnification and were analyzed using Zen 2 software. The area indicated by EC-positive staining or MBP + /GFAP + immunoreactivity was circled manually to show the spare tissue.

For analysis of cell densities, the epicenter of the spinal cord and the next two sections 200–400 μm rostral and caudal for each animal were imaged. We performed systematic uniform random sampling within each section by overlying a grid (individual grid size 110 μm × 110 μm) onto a low-magnification preview image of a cross section of the spinal cord. Then every 3 × 3 grids were imaged at 400 × magnification. Cells were counted at 400 × magnification when they were located in the spinal cord or touching the left and/or top of the box.

For analysis of the density of glial scar, the number of axons and myelin sheaths generated by SCs, sections, including epicenter and 200 and 400 μm rostral and caudal, were imaged at 400 × magnification for analysis. Similarly, grids were randomly overlaid within the sections at a low-magnification preview image; then the grids were taken images at 400× magnification. The number of axons and P0 + myelin sheaths, and the ratio of P0 + myelin sheaths to axons were calculated and compared between the two injured groups. In addition, the densities of GFAP + were calculated and compared among groups.

### Western Blot Analysis

The proteins were separated on a 6%/12%/15% sodium dodecyl sulfate–polyacrylamide gel electrophoresis (SDS-PAGE) gel and then electrotransferred onto a polyvinylidene difluoride membrane (Millipore). The membranes were blocked with Tris-buffered saline containing 0.1% Tween-20 and 5% non-fat dry milk for 1 h at RT. Thereafter, different primary antibodies, including rabbit anti-LC3B antibody (1:500; Cell Signaling Technology), rabbit anti-Beclin-1 antibody (1:1,000; Cell Signaling Technology), rabbit anti-p62 antibody (1:1,000, Cell Signaling Technology), rabbit anti-GAPDH antibody (1:10,000; Abcam), and mouse anti-β-tubulin (1:5,000, FDbio Science, Hangzhou, China) were used to incubate the membranes overnight at 4°C. After washing three times, the membranes were incubated by horseradish peroxidase-labeled secondary antibody (1:5,000; Beyotime) for 1 h at RT. Immunoblot was detected with the enhanced chemiluminescence (ECL) substrate (Bio-Rad) and digitized by GelView 6000Pro (BLT, Guangzhou, China). Band densities were quantified using the ImageJ 1.52 software program (National Institutes of Health). The quantities of the band densities were normalized to a loading control GAPDH or β-tubulin and then compared among the three groups.

### Statistical Analysis

The data in this article were expressed as mean ± standard deviation (SD). GraphPad Prism (Version 8.0, CA) was used for the statistical analysis. Unpaired *t*-test was used to analyze biomechanical parameters of contusion and immunostaining of P0 and Olig2 between the rapamycin and vehicle groups. One-way ANOVA followed by Turkey’s test *post hoc* was used to analyze the results of WB among the three groups. For EC staining and immunostaining densities of GFAP, NeuN, and MBP analyses, a two-way repeated-measures ANOVA was conducted with comparisons using Tukey’s *post hoc* to compare individual groups. Two-way ANOVA with repeated measurement was used to analyze behavioral assessment and weight change, and Dunnett’s/Sidak’s test was used for multiple comparisons.

## Results

### Biomechanical Parameter Analysis of Contusion

**FIGURE 1:**
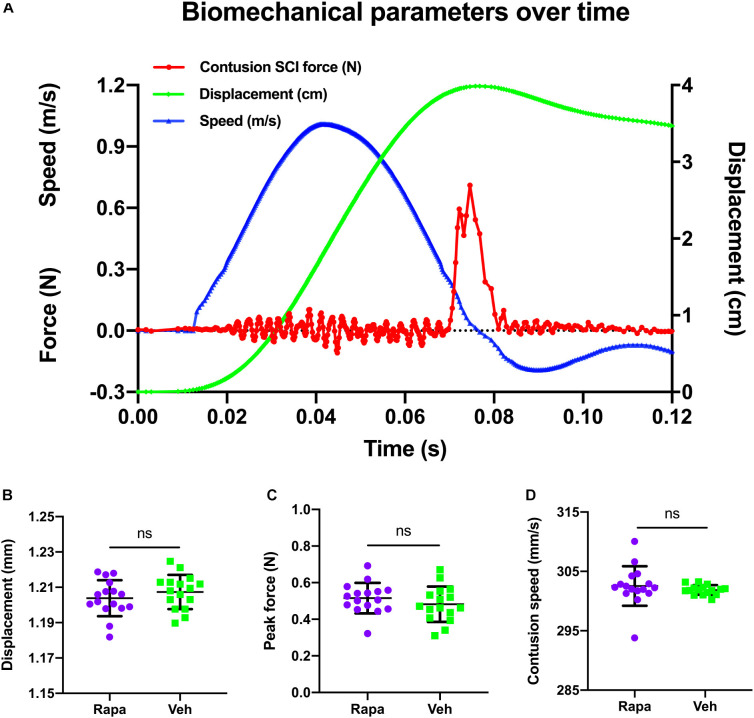
A representative graph of the changes of biomechanical parameters during contusion and their comparison in different groups. **(A)** Changes in biomechanical parameters over time. Red, contusion force; green, contusion displacement; blue, contusion speed. **(B–D)** There were no significant differences between the two injured groups in contusion displacement, contusion speed, and peak force. m/s, meters per second; N, newton; cm, centimeter; s, seconds; mm, millimeters; mm/s, millimeters per second. Rapa, the rapamycin-treated group; Veh, the vehicle-treated group. ^*ns*^*P* > 0.05. Error bars are mean ± SEM.

The changes of biomechanical parameters over time during contusion are shown in Figure 1A. The mean contusion displacement was 1.20 ± 0.01 mm in the rapamycin group and 1.21 ± 0.01 mm in the vehicle group (Figure 1B). Additionally, the contusion speed was 303 ± 3 mm/s in the rapamycin group and 302 ± 1 mm/s in the vehicle group (Figure 1C). The peak force was 0.48 ± 0.10 N in the rapamycin group and 0.52 ± 0.08 N in the vehicle group (Figure 1D). There was no significant difference between the two groups in contusion displacement, speed, or peak force (Figures 1B–D).

### Autophagy Was Enhanced by Rapamycin

**FIGURE 2:**
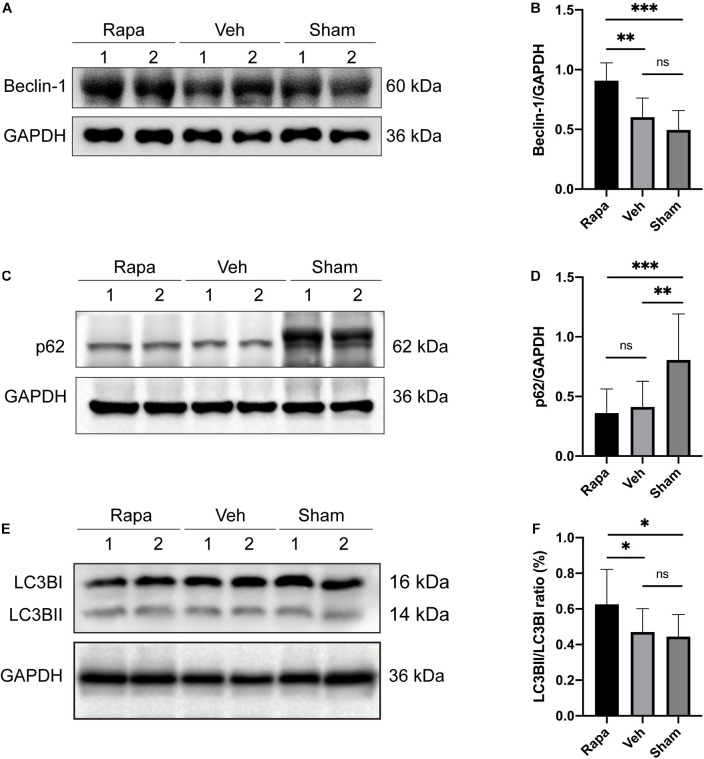
The representative Western blot images and the histogram showed the relative expression of Beclin-1, LC3BII, and p62. Representative Western blots and quantitative graphs demonstrate that the expression of Beclin-1 in the rapamycin-treated group was significantly greater than that in the vehicle and sham groups **(A,B)**, the expression of p62 decreased significantly in the two injured groups compared with the sham group after spinal cord injury (SCI) **(C,D)**, and the expression of LC3BII in the rapamycin group was significantly greater than that in the vehicle and sham groups **(E,F)**. Rapa, the rapamycin group; Veh, The vehicle group; Sham, the sham group. ^∗^*P* < 0.05, ^∗∗^*P* < 0.01, ^∗∗∗^*P* < 0.001, ^ns^*P* > 0.05. Error bars are mean ± SEM.

The expression levels of autophagy-related proteins in the spinal cord were assessed by WB to evaluate the effect of rapamycin treatment on autophagy after SCI (Figure 2). Compared with the sham group, the expression level of p62 was decreased significantly, and the expressions of Beclin-1 and LC3BII were slightly increased in the vehicle-treated group (Figures 2A–F). In addition, compared with the sham group, rapamycin significantly increased the expression of Beclin-1 and LC3BII and significantly decreased the expression of p62 in the rapamycin-treated group (Figures 2A–F). The expressions of Beclin-1 and LC3BII were increased significantly, and the expression of p62 slightly decreased in the rapamycin-treated group compared with the vehicle-treated group (Figures 2A–F).

### Rapamycin Reduced White Matter Loss After Spinal Cord Injury

**FIGURE 3:**
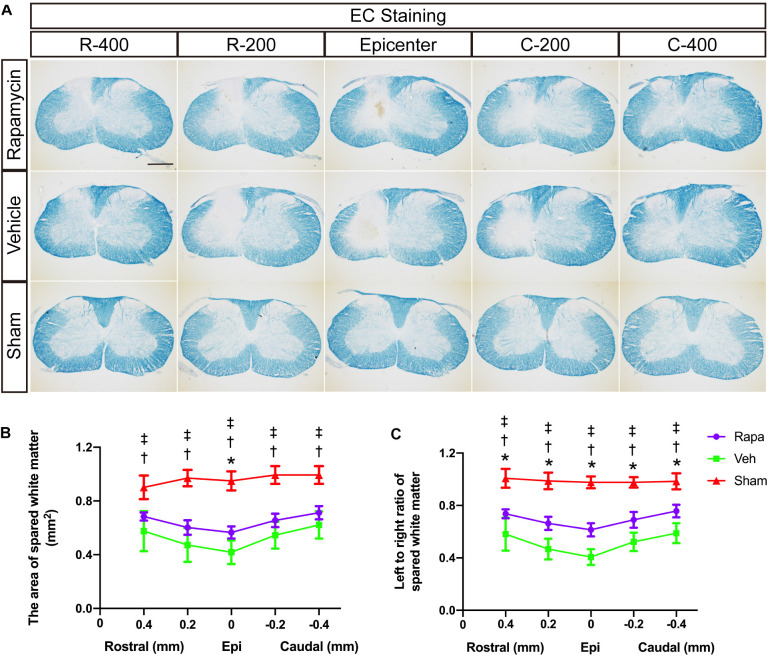
Eriochrome cyanine (EC) staining of the rostral–caudal spread of the lesions shows that more severe lesions extend for considerable distances in the spinal cord and affect white matter areas. **(A)** Histological sections processed for EC are shown for the three groups at the lesion epicenter and at 0.2 and 0.4 mm rostral and caudal. The graphs in **(B,C)** show the area of spared white matter of the left side and quantification of the left to right ratio at all intervals. Rapa, the rapamycin group; Veh, the vehicle group; Sham, the sham group; L, left; R, right; Epi, epicenter; mm, millimeters. *P < 0.05 between the rapamycin-treated and vehicle-treated groups; ^†^*P* < 0.05 between the rapamycin-treated and sham groups; ^‡^*P* < 0.05 between the vehicle-treated and vehicle-treated groups.

We also evaluated the effect of rapamycin on white matter after SCI. EC staining was used to stain myelin lipids. White matter loss was obviously observed at the epicenter and 0.2 and 0.4 mm rostral and caudal sides, which were mainly located on the ipsilateral side of the sections (Figure 3A). The areas of spared white matter in the rapamycin-treated group were significantly greater than those in the vehicle-treated group at the epicenter (0.57 ± 0.04 vs. 0.42 ± 0.09 mm^2^, *P* = 0.0182), but there were no differences at the 0.2 and 0.4 mm rostral and caudal (rapamycin vs. vehicle: 0.68 ± 0.03 vs. 0.58 ± 0.15 mm^2^ at 0.4 mm rostral, 0.60 ± 0.05 vs. 0.47 ± 0.13 mm^2^ at 0.2 mm rostral, 0.66 ± 0.05 vs. 0.55 ± 0.10 mm^2^ at 0.2 mm caudal, 0.71 ± 0.05 vs. 0.62 ± 0.10 mm^2^ at 0.4 mm caudal, respectively) (Figure 3B). In addition, there were significant differences in the ratio of spared white matter on the left to right among the three groups at each segment (Figure 3C).

**FIGURE 4:**
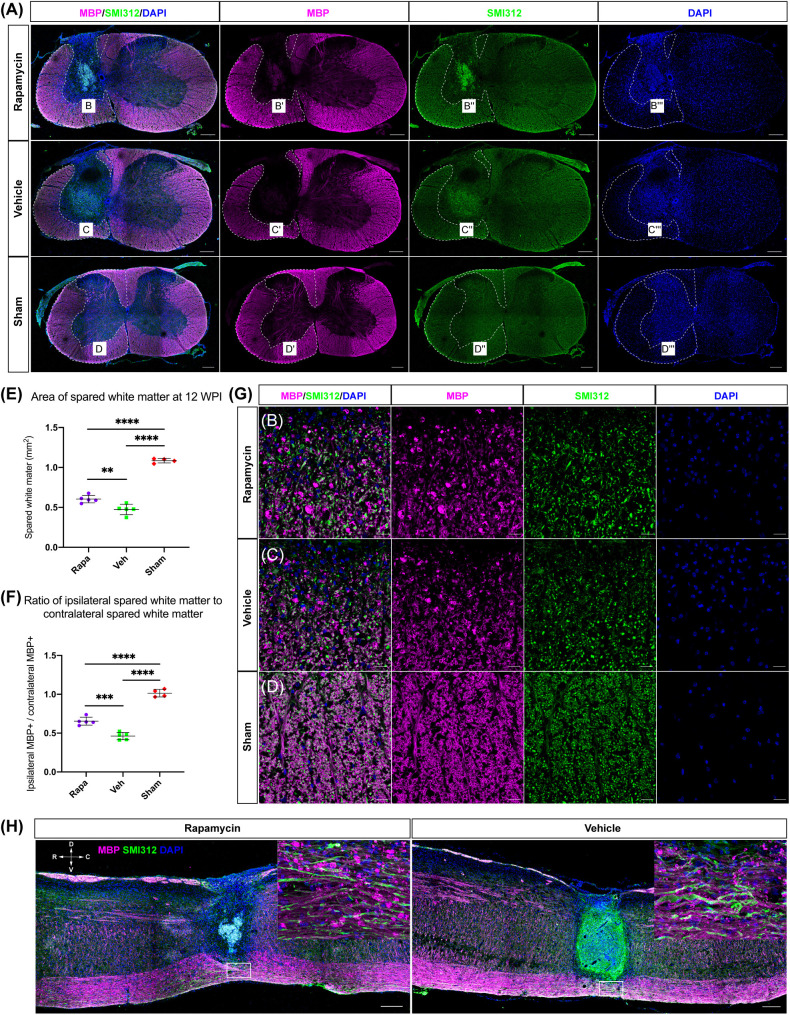
The area of spared white matter after injury. Demyelination was mainly located on the ipsilateral side at the injury epicenter [**A**, red: myelin basic protein (MBP), labeled myelin sheathes; green: SMI312, labeled axons; blue: DAPI, labeled nucleus]. High magnifications of demyelination are shown in **(B–D,G)**. The area of spared white matter was significantly decreased in the two injured groups, and the area of spared white matter in the rapamycin-treated group was significantly greater than that in the vehicle-treated group **(E,F)**. Longitudinal sections are shown in **(H)**. WPI, weeks post-injury; R, rostral; C, caudal; D, dorsal; V, ventral. ^∗∗^*P* < 0.01, ^∗∗∗^*P* < 0.001, ^*⁣*⁣**^P < 0.0001. Scale bar = 200 μm **(A–H)** and 20 μm **(B–D)**. Error bars are mean ± SEM.

MBP was also used to label the spared myelin to indicate the area of spared white matter (Figure 4A). Within the spared white matter, numerous myelin debris was observed in the two injured groups (Figures 4B–D). Because a hemi-contusion injury model was used in the present study, the areas of spared white matter on the ipsilateral and contralateral sides were analyzed separately. The contusion injury produced a significant myelin loss at the ipsilateral spinal cord in the injured mice (Figure 4A), and the area of spared white matter was significantly decreased in rapamycin-treated and vehicle-treated groups compared with the sham group (Figure 4E). Also, the area of spared white matter was significantly greater in the rapamycin-treated mice than in the vehicle-treated mice at 12 WPI (Figure 4E).

The ratio of ipsilateral white matter to contralateral white matter was calculated, and the area of ipsilateral spared white matter in the rapamycin-treated group was significantly greater than that of the vehicle-treated group, indicating that the area of ipsilateral white matter was sharply decreased after contusion injury (Figure 4F). In addition, the longitudinal section at the epicenter of the spinal cord showed the lesion area in the vehicle-treated group was larger than that in the rapamycin-treated group (Figure 4H).

### Rapamycin Promotes Schwann Cell-Mediated Remyelination After Spinal Cord Injury

**FIGURE 5:**
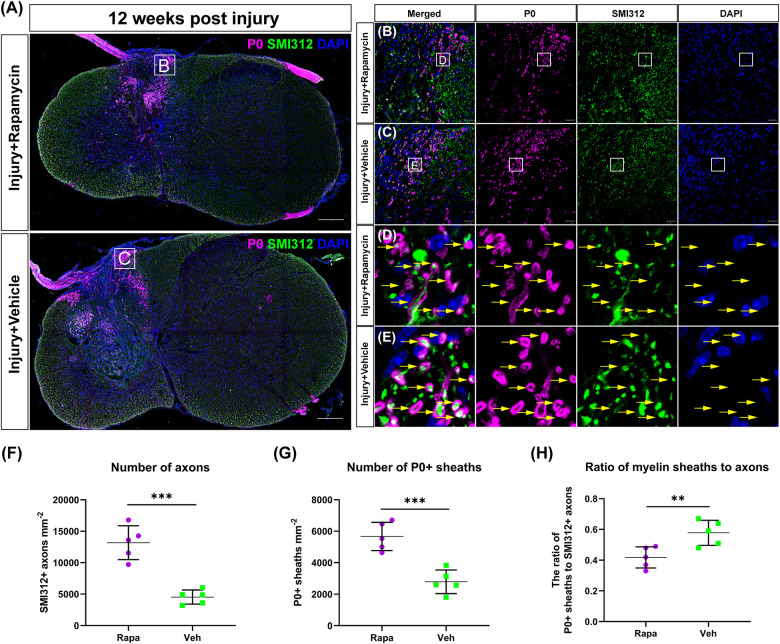
The Schwann cell-mediated remyelination at the injury epicenter of the two injured groups at 12 weeks post-injury. Immunofluorescent staining was used to detect the myelin formed by Schwann cells, and the P0 + myelin was observed around the lesion site and at the nerve roots **(A)**. High-magnification images of **(A)** are shown in **(B,C)**. **(D,E)** The representative images of the P0 + myelin (yellow arrows). Quantitation of the SMI312+ axons, P0 + myelin, and the ratio of myelin sheaths to axons were shown in **(F–H)**, and SMI312 + axons and P0 + myelin sheaths were significantly increased in the rapamycin-treated mice when compared with the vehicle-treated mice. The ratio of P0 + myelin to SMI312 + axons in the rapamycin-treated mice was significantly less than that in the vehicle-treated mice. **P < 0.01, ***P < 0.001. Scale bar = 200 μm **(A)** and 20 μm **(B,C)**. Error bars are mean ± SEM.

To investigate the effect of rapamycin on Schwann cell-mediated remyelination at the epicenter after SCI, we specifically labeled the myelin sheath with the myelin protein zero (P0) and analyzed its fluorescence density. At 12 WPI, myelin formed by SCs was observed at the epicenter in both two injured groups, and the P0 + myelin was located in the periphery of the lesion and the nerve root (Figure 5A). Axons were wrapped by P0 + myelin in both the rapamycin-treated group and vehicle-treated group, and only the P0 + myelin located in the spinal cord was used for analysis (Figures 5B–E). The results showed that the SMI312 + axons and P0 + myelin sheaths in the rapamycin-treated mice were significantly greater than those in the vehicle-treated mice, respectively (Figures 5F,G), while the ratio of P0 + myelin to SMI312 + axons in the rapamycin-treated mice was significantly less than that in the vehicle-treated mice (Figure 5H).

### Rapamycin Contributed to the Accumulation of Oligodendrocytes After Spinal Cord Injury

**FIGURE 6:**
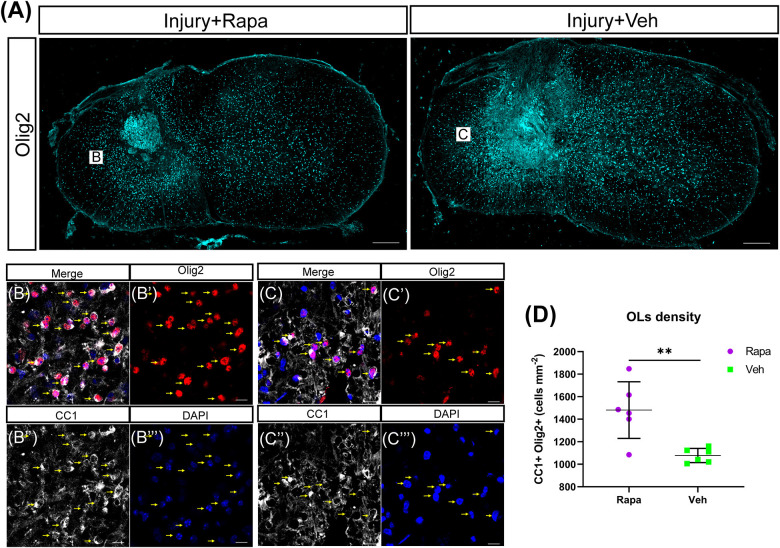
The number of oligodendrocytes (OLs) was increased in the rapamycin-treated mice at 12 weeks post-injury. **(A)** Overview of Olig2 staining at injury epicenter in the rapamycin-treated and vehicle-treated groups. Boxes are approximate areas where **(B,C)** were imaged. **(B,C)** High-magnification representative images of the lateral white matter stained with Olig2 and CC1 in the rapamycin-treated and vehicle-treated mice. Yellow arrows in **(B,C)** indicate OLs. **(D)** Quantification demonstrates that the rapamycin-treated mice have a higher density of OLs (Olig2 + CC1 +) than the vehicle-treated mice. Rapa, the rapamycin group; Veh, the vehicle group. ^∗∗^*P* < 0.01. Scale bars = 200 μm **(A)** and 10 μm **(B,C)**. Error bars are mean ± SEM.

We used CC1 and Olig2 to co-label the mature OLs and observed their changes in the rapamycin-treated and vehicle-treated mice. OL lineage cells (Olig2 +) were mainly located in the spared spinal cord tissue rather than in the lesion site (Figure 6A). A lot of OLs (CC1 + Olig2 +) were observed at 12 WPI (Figures 6B–E). The number of OLs was increased significantly in the rapamycin-treated group compared with the vehicle-treated group (Figure 6F).

### Rapamycin Reduced Astrocyte Activation and Glial Scar Formation

**FIGURE 7:**
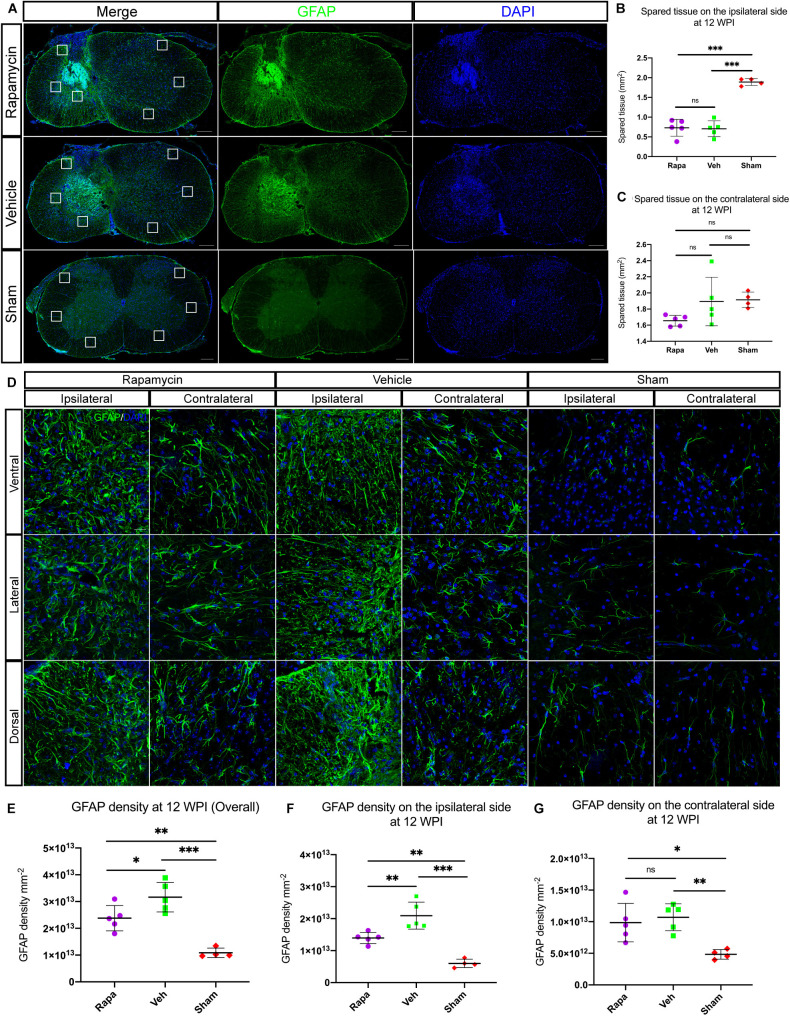
Rapamycin alters the activation of astrocytes and glial scar formation at the epicenter of spinal cord after injury. **(A)** Astrocytes were activated, and glial scar was formed at the epicenter after injury. The areas of spared tissues indicated by glial fibrillary acidic protein (GFAP) + on the ipsilateral side were significantly decreased in the two injured groups compared with the sham group **(B)**, while the spared tissue on contralateral side showed no significant differences among the three groups **(C)**. Representative high-magnification images on the ipsilateral and contralateral sides of the spinal cord are shown in **(D)**. Quantitation of GFAP densities showed that GFAP densities were significantly increased in the two injured group compared with the sham group after spinal cord injury (SCI) **(E–G)**. There were significant differences in GFAP density of the whole spinal cord and the ipsilateral side between the rapamycin and vehicle groups **(E,F)**, but there was no significant difference in GFAP density of the contralateral side between the two groups **(G)**. WPI, weeks post-injury; Rapa, the rapamycin group; Veh, the vehicle group; Sham, the sham group. Scale bar = 200 μm **(A)** and 20 μm **(D)**. ^∗^*P* < 0.05, ^∗∗^*P* < 0.01, ^∗∗∗^*P* < 0.001, ^*ns*^*P* > 0.05. Error bars are mean ± SEM.

The glial scar was one of the influential factors affecting the recovery of limb function. Therefore, we analyzed the effects of rapamycin on glial scar formation. The area of spared tissues indicated by GFAP + was measured, and the result showed that the area of spared tissues on the ipsilateral side of the two injured groups was significantly reduced than of the sham group, whereas on the contralateral side, there were no significant differences among the three groups (Figures 7A–C). Additionally, GFAP + immuno-densities on the entire section of the spinal cord were analyzed. Overall, GFAP density was significantly different among the three groups (Figures 7D,E). Because we used a model of hemi-contusion injury, we also analyzed the GFAP + density on the ipsilateral side and contralateral side. GFAP density was significantly increased at the epicenter on both sides of the spinal cord in the two injured groups compared with the sham group (Figures 7F,G). On the ipsilateral side, GFAP density was significantly decreased in the rapamycin-treated mice compared with the vehicle-treated mice (Figure 7F). On the contralateral side, however, there was no significant differences among the three groups (Figure 7G).

### The Effect of Rapamycin on Residual Neurons After Spinal Cord Injury

**FIGURE 8:**
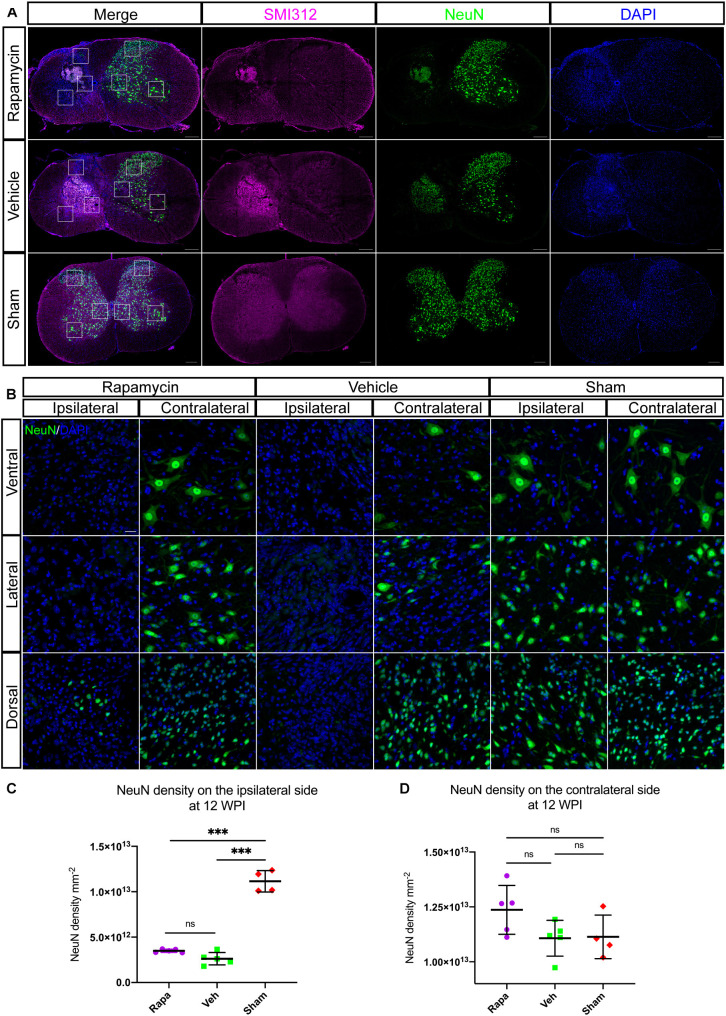
Rapamycin does not alter the neuronal damage after spinal cord injury (SCI). Immunostained sections of the injury epicenter of the spinal cord with NeuN + and SMI312 + are shown in **(A)**. High-magnification images of the NeuN + cells on the ipsilateral and contralateral side are shown in **(B)**. **(C)** The NeuN + cells were significantly decreased on the ipsilateral side at the injury epicenter in the rapamycin and vehicle groups. **(D)** Expression of NeuN on the contralateral side at the injury epicenter showed no significant differences among the three groups. WPI, weeks post-injury; Rapa, the rapamycin group; Veh, the vehicle group; Sham, the sham group. Scale bar = 200 μm **(A)** and 20 μm **(B)**. ^∗∗∗^*P* < 0.001, ^*ns*^*P* > 0.05. Error bars are mean ± SEM.

To investigate the role of rapamycin in NeuN-positive cells after hemi-contusion SCI, we calculated the number of residual neurons on the ipsilateral and contralateral sides. The NeuN-positive cells were mainly located on the gray matter, and there were few residual neurons on the ipsilateral side of the spinal cord after hemi-contusion injury (Figure 8A). The number of ipsilateral NeuN-positive cells of the vehicle-/rapamycin-treated mice was significantly decreased than that of the sham group at 12 WPI (Figures 8B,C), and no significant difference in the number of ipsilateral NeuN-positive cells was observed between the two injured groups (Figure 8C). On the contralateral side, there was also no significant difference in the number of NeuN-positive cells among the three groups (Figure 8D).

### Behavioral Assessment and Body Weight

**FIGURE 9:**
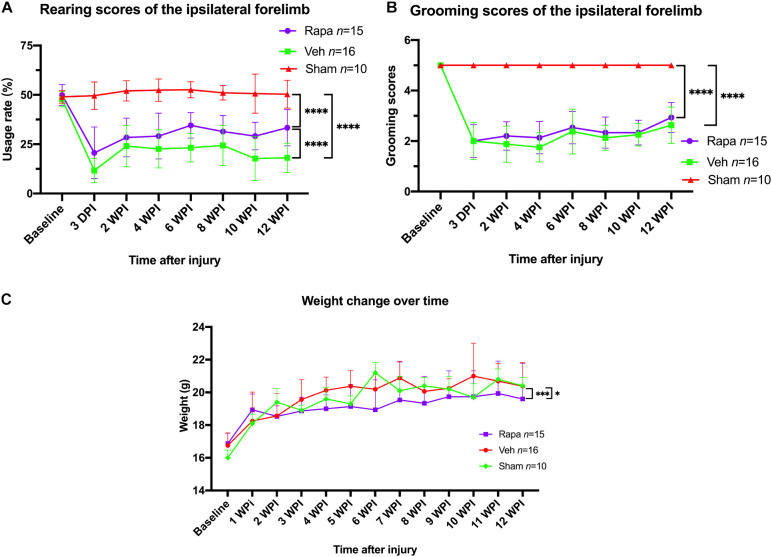
The changes in behavioral scores and body weight with time. **(A)** Rearing scores showed that rapamycin can increased the usage rate of the ipsilateral forelimb after spinal cord injury (SCI). **(B)** Grooming scores showed that the function of elbow and shoulder on the ipsilateral side was not improved by rapamycin after SCI. **(C)** The administration of rapamycin decreased body weight gain after injury. Rapa, the rapamycin group; Veh, the vehicle group; Sham, the sham group; WPI, weeks post-injury. ^∗^*P* < 0.05, ^∗∗∗^*P* < 0.001, ^*⁣*⁣**^P < 0.0001. Error bars are mean ± SEM.

Mice behavior changes were analyzed through a rearing test, which reflected the utilization of forelimb after injury. The preoperative rearing score was 49.9 ± 5.3 in the rapamycin-treated group and 47.7 ± 4.0 in the vehicle-treated group (Figure 9A). After SCI, the rearing scores in the rapamycin-treated group were 20.6 ± 13.1, 28.4 ± 9.8, 29.1 ± 11.6, 34.5 ± 6.5, 31.1 ± 8.4, 29.1 ± 7.2, and 32.4 ± 8.8 on postoperative day 3, week 2, week 4, week 6, week 8, week 10, and week 12, respectively; the rearing scores in the vehicle group were 12.4 ± 5.5, 24.7 ± 0.6, 23.0 ± 9.9, 23.4 ± 7.4, 25.0 ± 10.2, 18.4 ± 11.3, and 18.0 ± 7.7 on postoperative day 3, weeks 2, 4, 6, 8, 10, and 12, respectively (Figure 9A). The utilization rates of the ipsilateral forelimb in the two injured groups were significantly lower than the baseline and that in the sham group after injury (Figure 9A). At 12 WPI, the utilization of the ipsilateral forelimb in the rapamycin-treated group was significantly greater than that in the vehicle-treated group (Figure 9A).

For the grooming test, the ipsilateral grooming scores in the rapamycin group were 2.0 ± 0.7, 2.2 ± 0.6, 2.1 ± 0.6, 2.5 ± 0.6, 2.3 ± 0.6, 2.3 ± 0.5, and 2.0 ± 0.7 on postoperative day 3, weeks 2, 4, 6, 8, 10, and week 12, respectively, the ipsilateral grooming scores in the vehicle group were 2.1 ± 0.7, 1.9 ± 0.7, 1.8 ± 0.6, 2.5 ± 0.8, 2.1 ± 0.5, 2.2 ± 0.4, and 2.7 ± 0.7 on postoperative day 3, weeks 2, 4, 6, 8, 10, and 12, respectively (Figure 9B). The grooming scores of the sham group and the contralateral forelimb in the injured groups were 5.0, respectively (Figure 9B). The result showed that the ipsilateral grooming scores were significantly decreased in the two injured groups compared with the sham group after SCI (Figure 9B), and there was no significant difference in grooming scores between the rapamycin and vehicle groups after SCI (Figure 9B).

The mice body weight was monitored weekly pre-operation and post-operation. Overall, body weight increased over time in all groups (Figure 9C). The body weight in the rapamycin-treated group increased slowly, and the body weight in the rapamycin-treated mice was significantly less than that in the vehicle-treated mice and sham groups (Figure 9C).

## Discussion

In the present study, we demonstrated that rapamycin administration promotes the recovery of motor function and alleviates myelin loss after SCI. We regulated autophagy by performing a pharmacological method, rapamycin, to demonstrate the role of autophagy in SCI. Rapamycin enhanced autophagy, resulting in higher expressions of Beclin-1 and LC3 and lower expressions of p62, as well as significant improvement of motor function after SCI. Furthermore, the rapamycin treatment increased the area of spared white matter at 12 WPI and also reduced the glial scar formation and the loss of spinal cord tissues. What is more, the rapamycin treatment increased the number of axons and SC-associated myelin sheaths and decreased the body weight of mice after SCI. Our study indicated that rapamycin treatment is neuroprotective on motor function and white matter of the spinal cord after SCI.

Numerous studies have shown that rapamycin can enhance autophagy through pharmacological action and improve hindlimb function (Sekiguchi et al., 2012; Chen et al., 2013). And several reports indicated that rapamycin exerted neuroprotective effects after SCI by reducing inflammatory reactions (Goldshmit et al., 2015; Song et al., 2015; Tateda et al., 2017), decreasing neural tissue damage (Sekiguchi et al., 2012), attenuating glial scarring (Goldshmit et al., 2015), and increasing neuronal survival (Goldshmit et al., 2015). However, Hu et al. indicated that inhibition of autophagy through pharmacological effects can promote motor function recovery after SCI in rats (Hu et al., 2010), which is inconsistent with previous studies. In the present study, we demonstrated that the administration of rapamycin after hemi-contusion SCI had neuroprotective effects, including alleviating myelin loss, reducing glial scar formation, and promoting the recovery of motor function. And we also observed the enhancement of autophagy. However, no causal relationship between autophagy and neural function has been further demonstrated by inhibiting autophagy. Furthermore, Saraswat Ohri et al. (2018) used genetic methods to further indicate that blocking autophagy of OLs can hinder functional recovery after SCI. Therefore, we believed that the improvement of neural function may be induced by the enhancement of autophagy.

White matter loss can be observed at the epicenter of the spinal cord after SCI (Rowland et al., 2008; Mondello et al., 2015; Zhang et al., 2016), as reflected by the loss of myelin sheaths. The aggregation of inflammatory cells, the release of inflammatory factors, and the phagocytosis of myelin debris by macrophages are involved in the myelin loss process (George and Griffin, 1994; Gensel and Zhang, 2015; Song et al., 2015; Tateda et al., 2017). In the present study, the area of spared white matter in the rapamycin-treated mice was significantly greater than that in the vehicle-treated mice, which may be caused by decreased myelin loss and/or increased remyelination. And the number of mature OLs was increased significantly in the rapamycin-treated group, which has a close relationship with the formation of myelin sheaths. In addition, unmyelinated axons were still observed at the edge of the lesion after injury. However, due to the lack of genetic fate mapping new myelin and the results of an electron microscope, it was difficult to distinguish whether these myelin sheaths were new or residual in this study. Previous studies showed that macrophages were activated and secrete a large number of inflammatory cytokines after the injury (Nocentini et al., 2008; Sato et al., 2012), which will lead to OLs death and myelin sheaths loss (George and Griffin, 1994; Nave and Trapp, 2008; Almad et al., 2011; Plemel et al., 2014). And inflammation after SCI was also affected by the pathways that were regulated by mTOR (Goldshmit et al., 2015; Tateda et al., 2017). Therefore, we speculate that rapamycin may reduce inflammation after SCI by enhancing autophagy (Goldshmit et al., 2015; Tateda et al., 2017), which helps restore OLs and myelin sheaths to limit the axonal degeneration. These were closely related to the recovery of motor function after SCI. Clearly, further research is needed to provide sufficient evidence.

At the same time, these pathological processes promoted the proliferation of OL progenitor cells (OPCs), which can differentiate into OLs and SCs (Levine and Reynolds, 1999; Rhodes et al., 2006; Nakatani et al., 2013; Assinck et al., 2017) and then form new myelin sheaths in the CNS (Tripathi et al., 2010; Zawadzka et al., 2010). The myelin sheaths generated in remyelination are thinner and shorter than the original sheaths (Blakemore, 1974), indicating that endogenous spontaneous myelin regeneration might be not sufficient to promote functional recovery after SCI. Our study showed a significantly higher number of OLs in the rapamycin-treated mice, which might lay the foundation for remyelination after SCI. A previous study demonstrated that autophagy participated in the development of OL lineage in the nerve system, while the deletion of *Atg5* (and hence autophagy) in adult mice had no effects on the integrity of myelin sheaths (Saraswat Ohri et al., 2018). Furthermore, the *Atg5* deletion decreases the proliferation and differentiation of OLs after SCI, and it is assumed that these were caused by the reduced autophagic flux. In addition, the pharmacologic target of autophagy, mTOR, was also involved in the development and maturation of OLs and SCs (Tyler et al., 2009; Gomez-Sanchez et al., 2015). Tyler et al. indicated that mTOR can regulate OL differentiation at the late progenitor to immature OL transition, showing that the activation of mTOR is essential for OL differentiation. They also demonstrated that mTOR1 and mTOR2 complexes affected the expression of MBP through different mechanisms. In the present study, the number of OLs was increased in the rapamycin-treated group, which was contrary to observation from the previous study. The reason may be due, in part, to rapamycin favoring OL survival to increase its number after SCI. Also, Saraswat Ohri’s study showed that the increased autophagic flux, which can be induced by rapamycin, might increase the proliferation and differentiation of OLs (Saraswat Ohri et al., 2018). Therefore, to promote endogenous myelin regeneration by regulating autophagy to protect the residual axons may be a strategy for promoting functional recovery after SCI.

The remyelination in the CNS can sometimes be mediated by SCs, which also contributed to the functional recovery (Honmou et al., 1996; Bartus et al., 2016, 2019). Previous studies showed that SCs presented in CNS after SCI were mainly derived from CNS-resident precursors, OPCs (Zawadzka et al., 2010; Assinck et al., 2017); and neuregulin-1 (Nrg1) was a key factor in the differentiation of OPCs after contusion SCI (Bartus et al., 2016). Nrg1 signals via ErbB tyrosine kinase receptors and activated PI3K/Akt pathway, leading to a further increase phosphorylation of mTOR protein and activation of mTOR signaling. A study showed that the specific ablation of ErbB receptor reduced the production of SCs after SCI, indicating that Nrg1/ErbB signaling can promote the transformation of OPCs into remyelinating SCs (Bartus et al., 2019). In other words, the deletion of Nrg1/ErbB signaling can affect the activation of mTOR and prevent the formation and remyelination of SCs after SCI. The above results were consistent with our observation that SCs produced a significantly increased number of myelin sheaths in mice treated with rapamycin. Rapamycin was a specific inhibitor of mTORC1, and autophagy has been shown to be involved in the development of SCs (Jang et al., 2015). Therefore, the downregulation of mTOR might induce OPCs to differentiate into OLs and SCs through enhancing autophagy. This conjecture was also supported by the OL increase in the rapamycin group in this study.

What is more, the number of axons was greater in the rapamycin-treated group than in the vehicle-treated group, which might be due to the effects of rapamycin on inflammation and glial scar after SCI. These resulted in that the ratio of myelin sheaths to axons of about 0.42 in the rapamycin-treated mice and 0.58 in the vehicle-treated mice, and there was a significant difference between the two groups. Schwann cell-mediated remyelination was increased in the rapamycin-treated group, and the motor function was significantly improved after injury, which was consistent with previous studies (Honmou et al., 1996; Bartus et al., 2016, 2019). Alternatively, this may also be due to autophagy enhanced by rapamycin had multiple effects on CNS after injury, including reducing inflammation and formation of astrocytes scar, and increasing neuronal survival, which were beneficial for the motor functional recovery. Therefore, targeting the differentiation of OPCs into SCs to form new myelin sheaths may be a potential therapeutic strategy for the treatment of SCI.

Another reason related to motor recovery may be that the glial scar formation was reduced (Tian et al., 2006; Li et al., 2016). Goldshmit et al. (2015) indicated that rapamycin decreased astrocyte reactivity at the lesion site after SCI through examining the levels of GFAP mRNA and the density of GFAP immuno-stained. Furthermore, Huang et al. (2017) demonstrated that rapamycin significantly inhibited the astrocyte activation induced by SCI. Our study showed that astrocyte reactivity of the rapamycin-treated group decreased significantly on the ipsilateral side than that of the vehicle-treated group, which was consistent with the results of previous studies. Attenuating glial scar formation is considered advantageous for axonal regeneration (Li et al., 2011; Cua et al., 2013), thereby restoring motor function, which is also one of the promising treatment strategies for SCI.

Previous studies reported side effects of rapamycin administration, such as elevating resting blood pressure and decreasing body weight after complete thoracic SCI in rats, as well as increasing the frequency of autonomic dysreflexia and elevating the absolute blood pressure induced by colorectal distention (Eldahan et al., 2018). Eldahan et al. (2018) demonstrated that the four-week rapamycin treatment prevented the normal increase in body weight in naïve and vehicle-treated rats and reduced the body weight significantly after SCI. In the present study, rapamycin treatment after SCI resulted in significant body weight loss in mice, which was consistent with the results reported in previous studies. The present study also demonstrated weight loss with rapamycin over a longer period of time. All these suggest that the pharmacological effect of rapamycin in preventing weight gain over time is systematic, with SCI exacerbating the weight loss.

There are several limitations in the present study. First of all, due to the imperfections of our electron microscope technique, the results of this study did not show the morphology of myelin more clearly. Secondly, the present study did not consider the experiment of autophagy inhibition after SCI, so the causal relationship between autophagy and the recovery of motor function could not be fully confirmed. Moreover, female mice were used in our study, and the influence of animal sex on the results of this study was not considered. In addition, this study only observed the treatment effect at a single time point under a severity of SCI, lacking longitudinal observation at multiple time points. Finally, targeted inhibition or promotion of OPC/OL autophagy should be considered to clarify the role of autophagy in demyelination and remyelination after SCI.

In conclusion, the administration of rapamycin enhanced autophagy after SCI. Our results demonstrated that rapamycin can alleviate white matter loss, reduce glial scar formation, increase recruitment of OLs, and promote SC-mediated remyelination, showing neuroprotection and potential regeneration of myelin in the nervous system after SCI. However, rapamycin had no significant impacts on the residual neurons after injury. More importantly, treatment with rapamycin after SCI can promote recovery of motor function, indicating a promising therapeutic strategy for the treatment of SCI.

## Data Availability Statement

The raw data supporting the conclusions of this article will be made available by the authors, without undue reservation.

## Ethics Statement

The animal study was reviewed and approved by the Experimental Animal Welfare and Ethics Committee of Southern Medical University.

## Author Contributions

JLiu and QZ conceived and designed the research. JLiu, RL, JLin, and ZuH performed the experiments. JLiu, RL, ZhiH, WJ, QL, XiuW, and XiaoW analyzed the data and wrote the manuscript. JLiu, RL, JLin, ZuH, ZhiH, QL, YY, and WJ obtained and made interpretation of the data. JLiu, HJ, and QZ read and approved the final draft. All the authors approved the final version of the article.

## Conflict of Interest

The authors declare that the research was conducted in the absence of any commercial or financial relationships that could be construed as a potential conflict of interest.
